# Mode of entry of a vaporized pyrethroid knockdown agent into the body of the housefly, *Musca domestica* (Diptera: Muscidae)

**DOI:** 10.1007/s13355-016-0443-2

**Published:** 2016-09-20

**Authors:** Yusuke Sumita, Hitoshi Kawada, Noboru Minakawa

**Affiliations:** 1 Graduate School of Biomedical Sciences, Infection Research, Nagasaki University, 1-12-4 Sakamoto, Nagasaki, Nagasaki 852-8523 Japan; 2 Department of Vector Ecology and Environment, Institute of Tropical Medicine (NEKKEN), Nagasaki University, Sakamoto 1-12-4, Nagasaki, 852-8523 Japan; 3 Health & Crop Sciences Research Laboratory, Sumitomo Chemical Company Limited, Takatsukasa 4-2-1, Takarazuka, Hyogo 665-8555 Japan

**Keywords:** Insecticide, Vapor action, Empenthrin, Spiracle, Central nervous system

## Abstract

We investigated the mode of entry of pyrethroids into the insect body using adult housefly, *Musca domestica* L., as an insect model. The wings of adult female houseflies were removed, and empenthrin was applied topically to three different sites: the mesothoracic spiracle, the ventral mesothorax, and the dorsal mesothorax. Among these treatments, the application of the compound to the mesothoracic spiracle led to the quickest knockdown of the flies. To determine the importance of the spiracle as a primary entry site for the pyrethroid, knockdown times were compared between houseflies with blocked and non-blocked spiracles, using two bioassays: a vapor action test using technical grade empenthrin, and a mosquito coil test using empenthrin-impregnated coils. In both tests, the times required for 50 % knockdown of spiracle-blocked houseflies were significantly higher than those required for the non-blocked flies. However, the mortality rates of the two groups were nearly identical, suggesting that spiracles play an important role in the knockdown of houseflies. These results also suggest that the rate of pyrethroid uptake through the spiracles was decreased due to the blocking of the mesothoracic spiracle. Therefore, the spiracle may be considered the main entry site for vaporized pyrethroids.

## Introduction

Insecticides are indispensable tools for the control of disease vectors and the improvement of public health (Mauro et al. 2012; WHO 2006). Studies investigating the modes of insecticide action and entry are critical for maximizing the efficiency of insecticide use. Several modes of entry have been reported, such as penetration through the integument, mouthparts, and spiracles. Previous studies have suggested that insecticides taken up by insects via physical contact pass through the integument into the body before being transported to the central nervous system (CNS) via the hemolymph (Ebeling 1974; Grissom et al. 1989; Lewis 1957; Matsumura 1963; Noble-Nesbitt 1970; Yu 2008). Fumigants such as hydrogen cyanide (HCN) and methyl bromide enter insects through the tracheal system, in tandem with carriers such as carbon dioxide (CO_2_) (Busvine 1971; Jones 1938). Gerolt (1969) speculated that carbon-14-dieldrin spread laterally across the integument on contact, and penetrated through the tracheae to the CNS via the hemolymph. Sugiura et al. (2008) suggested that the mesothoracic spiracles of the cockroach are one of the most effective entry routes for pyrethroids applied directly in the form of aerosols. Insect spiracles on the ventral or dorsal mesothorax could be a primary target for knockdown agents such as pyrethroids and dichlorodiphenyltrichloroethane, as they allow for rapid entry into the system and provide the quickest route to the CNS (Huber 1974). This leads to a quicker reaction (knockdown) than can be accomplished using other methods. However, the vapor’s mode of entry into the bodies of flying insects is not yet fully understood. Air passing through the spiracles enters the longitudinal tracheal trunks, spreads, and branches into the network of tracheal tubes before reaching every part of the body, including the CNS (Burrows 1980). If the vapor’s main entry site is the spiracle, the onset of knockdown can be delayed by blocking the spiracles. To investigate the main entry route of vaporized pyrethroids, we first identified the application point on the thorax that caused the fastest response in houseflies. Thereafter, we examined whether or not blocking the spiracles decreased the effectiveness of the insecticides.

## Materials and methods

### Insects

An insecticide-susceptible strain of housefly, *Musca domestica* L., obtained from the Chemical Specialties Manufacturers’ Association, USA, was used in this study. The strain was maintained in the laboratory at room temperature (25 ± 2 °C), 60–90 % relative humidity, and under a 14-h:10-h light:dark day/night cycle. All experiments were conducted with 3- to 7-day-old female houseflies. The adult females were reared in a cage (length 210 mm, width 280 mm, height 210 mm) consisting of a stainless steel frame and nylon mesh sides, and provided with water and food (skimmed milk powder and granulated sugar).

### Chemicals

Technical grade empenthrin (purity 98.0 %, vapor pressure at 20 °C 0.22 Pa; Sumitomo Chemical, Tokyo) was used in all tests, except the test conducted to confirm the side-effects of blocked spiracles on the efficacy of technical grade permethrin (purity 96.4 %, vapor pressure at 20 °C—cis-isomers 2.9 × 10^−6^ Pa, trans-isomers 9.2 × 10^−4^ Pa; Sumitomo Chemical).

### Test of topical application to three different sites

To identify the site conferring the greatest sensitivity to knockdown compounds, empenthrin was applied topically to three different sites on the female houseflies, the mesothoracic spiracle, ventral mesothorax, and dorsal mesothorax, using an automatic micro-applicator (Auto Micro applicator; Burkard Manufacturing, Rickmansworth, UK). A solution of acetone and empenthrin (10 mg/mL) was diluted to concentrations of 1 and 0.1 mg/mL. Adult female houseflies were anesthetized using CO_2_, and their wings removed with scissors (length 115 mm; no. 14; AS ONE, Osaka). After being allowed to recover from anesthesia (2 h), the flies were fixed to vacuum tweezers (P-100; Nitto Kohki, Tokyo), and 0.1 µL of the solution was applied to the three sites. Treatment with 10, 1, and 0.1 mg of the solution amounted to 1, 0.1, and 0.01 µg of empenthrin/insect, respectively. Following treatment, the houseflies were each placed into individual plastic containers (200 cm^3^), and the time required for knockdown was recorded, up to a maximum of 300 s. Each concentration was applied to ten houseflies. The control group was treated with 0.1 µL of acetone. Each assay was repeated twice.

### Bioassay with spiracle-blocked houseflies

After being anesthetized with CO_2_, the wings of adult female houseflies were removed with scissors, and a droplet of the adhesive agent, cyanoacrylate (Aron alpha; Toagosei, Tokyo), was applied to one or both sides of the mesothoracic spiracle using a stainless steel pin.

In order to elucidate the negative effects of spiracle blocking on insect survival and knockdown susceptibility, the survival rate after spiracle blocking was observed. In the survival test, mortality at 24 and 48 h after blocking one side of the mesothoracic spiracle or blocking both sides was recorded and compared to that of the non-blocked control group. Five houseflies were prepared per treatment group. Each assay was repeated six times.

Additionally, the insecticide susceptibility of spiracle-blocked houseflies was evaluated via the topical application test. In this test, 0.1 µL of three concentrations of the permethrin test solution (10, 1, and 0.1 mg/mL) was applied topically to the dorsal mesothorax. Five houseflies were prepared per group. Lethal doses with a 50 % mortality rate (LD_50_s) were compared to the mortality rate of the control group. Each assay was repeated four times. In each test, each housefly was each placed inside an individual plastic container (200 cm^3^) and covered with a 1-mm-mesh nylon net. Cotton wool soaked with a 5 % (w/v) sugar solution was placed on the nylon net to prevent the houseflies from desiccating or starving.

### Vapor bioassay using technical grade empenthrin

**Fig. 1:**
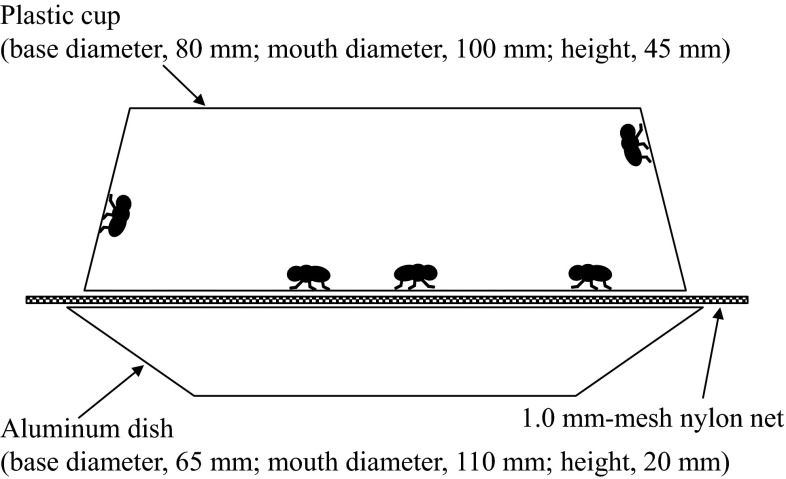
The container used for the vapor bioassay with technical grade empenthrin

The vapor bioassay, using technical grade empenthrin, was then conducted using houseflies whose spiracles had been blocked. The killing action of vaporized empenthrin was evaluated in aluminum busing dishes (Fig. 1). Ten milligrams of empenthrin was dissolved in 10 mL of acetone, and 0.7 mL of the solution was added to the aluminum dishes (base diameter 65 mm, mouth diameter 110 mm, height 20 mm) in a uniform layer, for a dosage of 100 mg active ingredient/m^2^. Treated dishes were dried for 30 min at room temperature. A plastic container (diameter 100 mm, height 45 mm) containing five house flies, the top of which was covered with a 1-mm-mesh nylon net, was turned upside down and placed on each treated dish to expose the insects to empenthrin vapor without direct contact (Fig. 1). The number of knocked down houseflies was counted each minute, for up to 20 min. The test was repeated four times using two groups of houseflies: one group with one side of the mesothoracic spiracles blocked, and one with non-blocked spiracles.

### Vapor bioassay using a mosquito coil

**Fig. 2:**
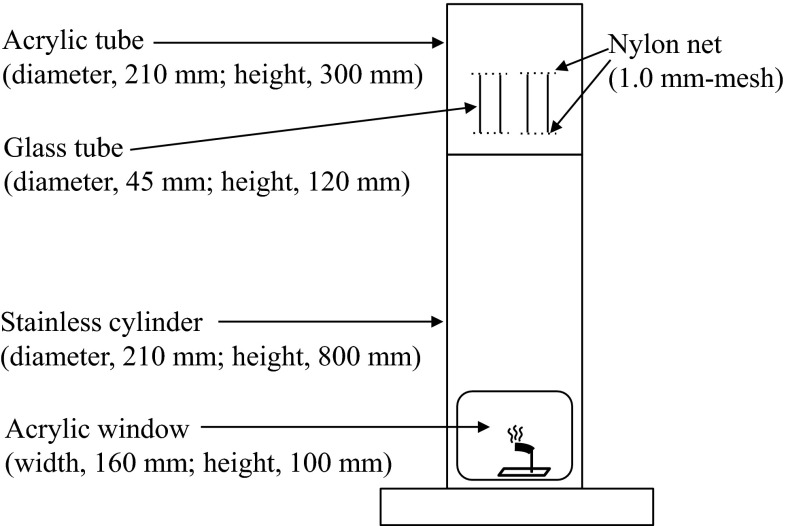
The cylinder used for the vapor bioassay with the mosquito coil formulation of empenthrin

The killing action of the empenthrin in a mosquito coil formulation was evaluated using a cylindrical, metal apparatus (Fig. 2). The solution of acetone and empenthrin (10 mg/mL) was diluted to obtain serial concentrations of 5, 3, and 1 mg/mL, respectively. One milliliter of each of these solutions was uniformly applied to 1-g pieces of the mosquito coil. In order to prepare the experimental mosquito coil pieces with 0.5, 0.3, 0.1 % empenthrin (w/w), the mosquito coils had been manufactured without insecticide (ingredients—pyrethrum mark, tabu powder, wood flour, malachite green, sodium dehydroacetate; Yamaguchi et al. 1981). The mosquito coil pieces were dried for 30 min at room temperature to remove the acetone. Five houseflies were placed in a glass tube (diameter 5 cm, height 12 cm), the tops and ends of which were covered with 1.5-mm-mesh nylon nets. Two tubes (one tube for the group with one side of the mesothoracic spiracle blocked and another tube for the group with non-blocked spiracles) were placed on the upper part of each cylinder at the same time, and ignited pieces of the mosquito coil were placed in the bottom of each metal cylinder (Fig. 2). The number of knocked down houseflies was counted each minute, for up to 20 min after the ignition of the mosquito coil. In addition, mortality was recorded at 24 h. The test was repeated five times with each of the two groups of houseflies.

### Statistical analysis

The LD_50_s of permethrin in the topical application test were calculated using Bliss’s probit method (Bliss 1934). Multiple comparisons of the mortality rates in the survival test among the three groups (one side blocked, both sides blocked, and non-blocked) were analyzed using the *χ* ^2^-test. The variations in the knockdown rates caused by topical applications and the vapor test were analyzed using Cox’s proportional hazards model. The time required for 50 % knockdown (KT_50_) in the topical application test with empenthrin and in the vapor test were estimated using the Kaplan–Meier survival analysis. Differences in mortality rates following exposure to empenthrin vapor were analyzed using the *χ* ^2^-test. Differences between the effects of mesothoracic spiracle blocking and wing removal on the susceptibility of houseflies to topically applied permethrin were analyzed using logistic regression. All statistical analyses except LD_50_s were conducted using the JMP 11 software package (SAS Institute, Cary, NC).

## Results

### Comparison of knockdown times in houseflies with empenthrin applied topically to three different sites

**Fig. 3:**
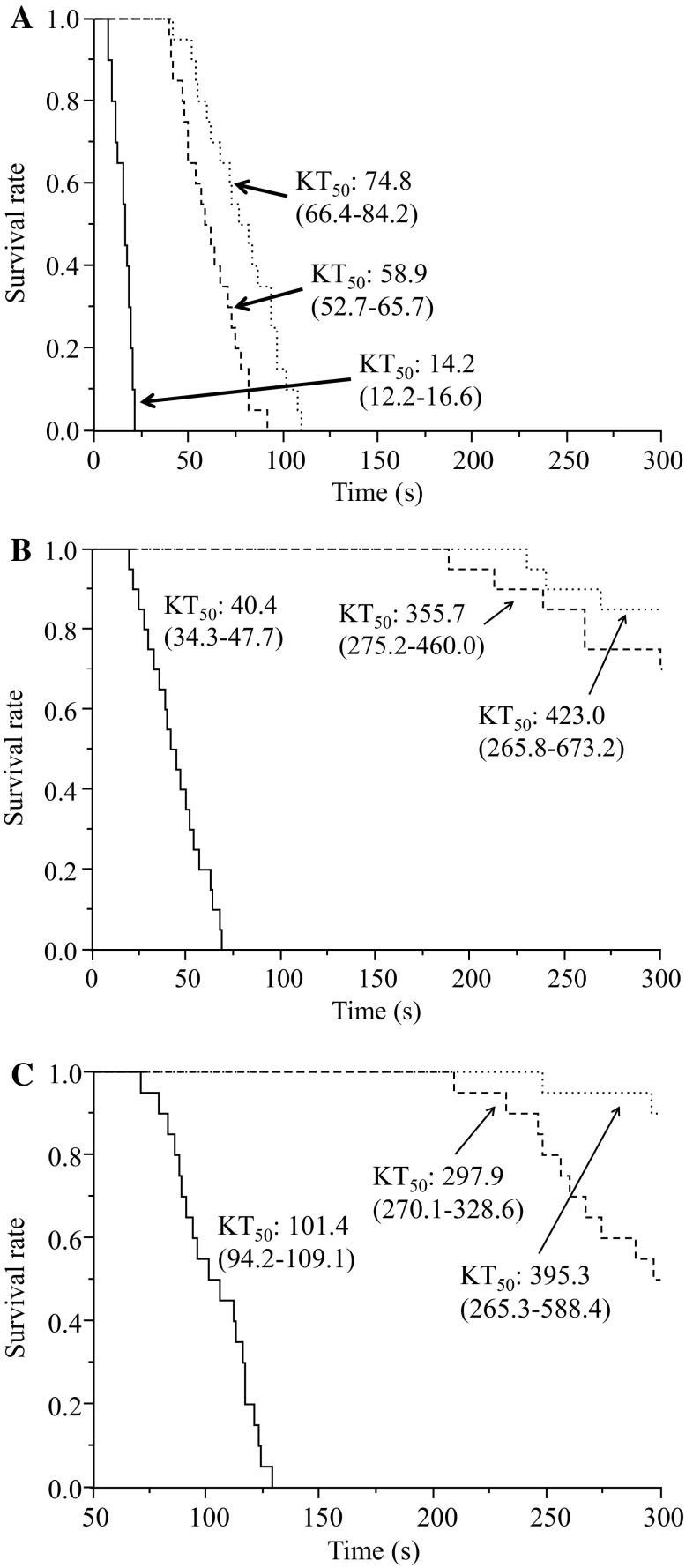
Knockdown rate of houseflies with mesothoracic (*solid line*), ventral mesothoracic (*dashed line*) and dorsal mesothoracic spiracles (*dotted line*) treated topically with empenthrin at 1.0 (**a**), 0.1 (**b**), and 0.01 µg (**c**) per female housefly. The 95 % confidence interval of the time required for 50 % knockdown (*KT*_50_) is shown* in parentheses*

We comparatively analyzed the knockdown times in houseflies treated with empenthrin on three different sites: Fig. 3 shows the KT_50_ values when empenthrin was applied to the mesothoracic spiracle, the ventral mesothorax, and the dorsal mesothorax at 1, 0.1, and 0.01 µg per female housefly. At all dosages, the KT_50_ values of the spiracle-treated group were lower than those of the other two groups. Cox’s proportional hazards model indicates that the knockdown activity was significantly affected by the empenthrin dosage (*df* = 1, *χ* ^2^ = 258.9, *p* < 0.0001) and the application point (*df* = 2, *χ* ^2^ = 248.0, *p* < 0.0001).

### Confirmation of negative effect of blocked spiracles

The mortality rates of the housefly group with one side of the mesothoracic spiracle blocked were 13.3 % at both 24 and 48 h, while the mortality rates of the housefly group with both sides of the mesothoracic spiracle blocked were 93.3 and 100 % at 24 and 48 h, respectively. The mortality rates of the housefly group with non-blocked spiracles were 10.0 and 16.7 % at 24 and 48 h, respectively (Table 1).

**Table 1:** Mortality rates of houseflies in the survival test after blocking their mesothoracic spiracles

Housefly group	*n*	Mortality after treatment (%)	
		24 h	48 h
One side of spiracle blocked	30	13.3 a	13.3 a
Both sides of spiracle blocked	30	93.3 b	100 b
Spiracle not blocked	30	10.0 a	16.7 a

*Shared alphabetical letters* in the* same column* indicate no significant difference according to *χ* ^2^-test with Bonferroni’s correction (*p* > 0.05)

*n* Number of females tested

At both points in time (24 and 48 h), the* χ* ^2^-test showed a significant difference in mortality rates among three groups (24 h, *df* = 2, *χ* ^2^ = 62.523, *p* < 0.0001, 48 h, *df* = 2, *χ* ^2^ = 72.568, *p* < 0.0001). The mortality rate of the housefly group with spiracles blocked on both sides was significantly higher (*p* < 0.0001 after Bonferroni correction).

Table 2 shows the LD_50_s of the houseflies with blocked mesothoracic spiracles treated topically with permethrin. The LD_50_s of the houseflies with one side of their mesothoracic spiracles blocked and wings removed, non-blocked spiracles and wings removed, and non-blocked spiracles and intact wings were 0.24, 0.21, and 0.22 µg per individual, respectively (Table 2). There was no significant differences in the LD_50_s (95 % confidence interval) among the three groups (Table 2).

**Table 2:** Effect of blocking the mesothoracic spiracle and the removal of wings on the susceptibility of houseflies to topically applied permethrin

Experimental group		*n*	LD_50_ value	95 % CI		Slope
Mesothoracic spiracle	Wings		(μg/female)			
One side blocked	Removed	40	0.24	0.21	0.28	5.5
Non-blocked	Removed	40	0.21	0.19	0.24	5.1
Non-blocked	Not removed	40	0.22	0.19	0.26	4.3

*n* Number of females tested,* LD*_50_ lethal dose of permethrin conferring a 50 % mortality rate,* CI* confidence interval

### Knockdown times for blocked and non-blocked houseflies in vapor bioassay with technical grade empenthrin

**Fig. 4:**
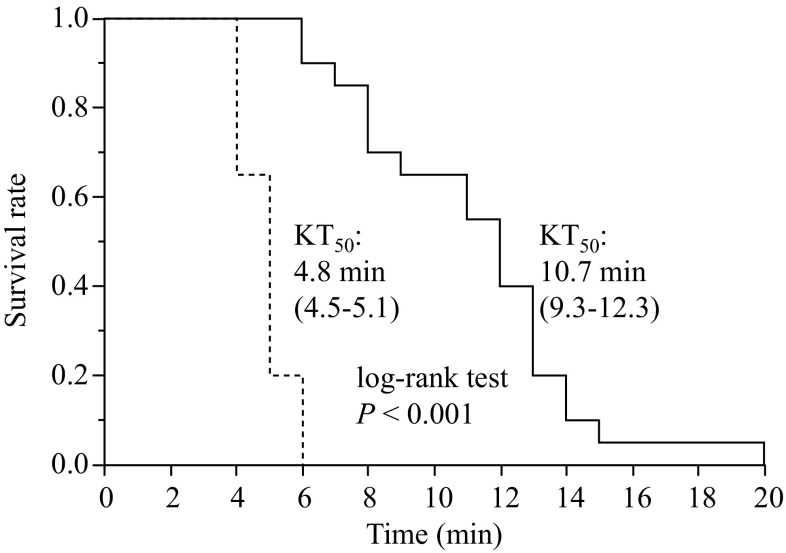
Knockdown rate of blocked-spiracle (*solid line*) and non-blocked-spiracle (*dashed line*) houseflies exposed to technical grade empenthrin in the form of a vapor. Survival curves are significantly different (log-rank test, *p* < 0.001, *n* = 20). The 95 % confidence intervals of the KT_50_ values are shown* in parentheses*

The KT_50_ values in the empenthrin vapor-treated blocked and non-blocked flies were 10.7 and 4.8 min, respectively (Fig. 4). The Kaplan–Meier survival analysis indicated that there was a significant difference between these two groups (*p* < 0.0001; log-rank test).

### Knockdown times of blocked and non-blocked houseflies in vapor bioassay using a mosquito coil

**Fig. 5:**
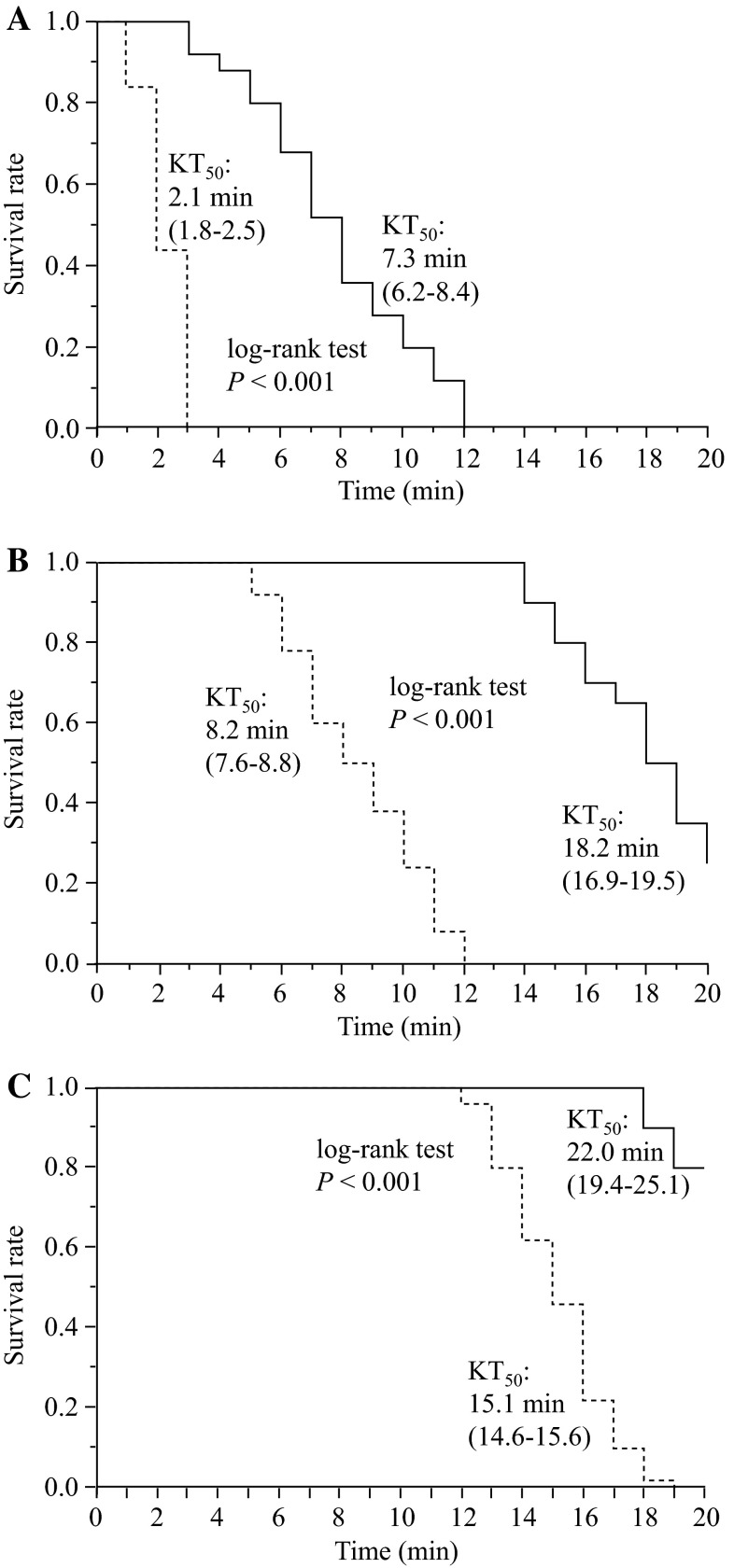
Knockdown rate of blocked-spiracle (*solid line*) and non-blocked-spiracle (*dashed line*) houseflies exposed to empenthrin vapor using a mosquito coil at 0.5 % (**a**), 0.3 % (**b**) and 0.1 % (**c**). Survival curves are significantly different (log-rank test, *p* < 0.001, *n* = 25). The 95 % confidence interval of the KT_50_ value is shown* in parentheses*

The KT_50_ values of the blocked and non-blocked houseflies were 7.3 and 2.1 min with a 0.5 % coil, 18.2 and 8.2 min with a 0.3 % coil, and 22.0 and 15.1 min with a 0.1 % coil, respectively (Fig. 5). Cox’s proportional hazards model (Table 3) indicated that the knockdown activity was significantly affected by the blocking of the spiracles (*df* = 1, *χ* ^2^ = 235.4, *p* < 0.0001), as well as by the concentration of empenthrin used (*df* = 2, *χ* ^2^ = 299.9, *p* < 0.0001). The KT_50_ values in houseflies with one side of their mesothoracic spiracles blocked, treated with 0.1, 0.3, and 0.5 % coils, were 1.5, 2.2, and 3.5 times greater, respectively, than those of the non-blocked houseflies. However, mortality rates at all concentrations were not significantly different, regardless of the presence or absence of blocked spiracles (*χ* ^2^-test, *p* > 0.05 in all concentrations; Table 3).

**Table 3:** Mortality rate of houseflies after exposure to empenthrin vapor from a mosquito coil

Spiracle	*n*	Concentration % (w/v)			
		0.1	0.3	0.5	Blank
Blocked	20	15	70	100	15
Non-blocked	20	25	80	100	20

*n* Number of females tested

## Discussion

The results suggest that the mesothoracic spiracles are primary entry sites for pyrethroids, and lead to the fastest knockdown response in houseflies. However, the application of pyrethroids to other parts of the mesothorax was less effective at causing the knockdown effect than application to the spiracles. Although it is not known whether, in this study, the insecticide applied to the other mesothorax sites reached the CNS through the integument or lateral spiracles, it is clear that a longer period is required for the insecticide to reach the CNS in cases where it is applied to sites other than the spiracles. Insecticide taken up through the spiracles seemed to rapidly reach the CNS through the longitudinal tracheal trunks directly connected to the CNS.

In the vapor bioassays using technical grade empenthrin or mosquito coils impregnated with the compound, knockdown occurred significantly slower in blocked-spiracle houseflies than in non-blocked-spiracle houseflies, suggesting that the amount of insecticide taken up through the spiracle per unit of time was reduced by the blocking of the mesothoracic spiracles. Interestingly, mortality rates were almost the same regardless of whether spiracles were blocked or not. Significant differences in knockdown times and non-significant differences in mortality rates between blocked and non-blocked houseflies might indicate that spiracles play a significant role in the knockdown process. The knockdown times between this experimental results with an additional experiment using non-vaporizing, slow-acting pyrethroids such as permethrin or phenothrin should be investigated.  Also, the knockdown times at different application sites such as spiracles and legs should be compared using vaporizing and non-vaporizing pyrethroids.

Several studies have investigated the mode of entry of insecticides. Dichlorvos and nicotine were found to enter through the insect cuticle, despite their high vapor pressures (Galley 1967). Matsumura (1963) found that a large amount of malathion was taken up from a glass surface through the legs of the American cockroach, suggesting that the tracheal system plays a minor role in its overall uptake. In these cases, the spiracles did not seem to be important for the uptake of insecticides such as organophosphates and nicotines. However, CO_2_ is often used as a carrier to increase the insecticidal efficacy of some fumigants that include nicotine, dichlorvos, and HCN, as it enhances the penetration of the tracheal system by insecticides (Bond 1961; Busvine 1971; Jones 1938; Terriere 1982). This indicates that the main mode of entry of insecticides might depend on the formulation used.

Gerolt (1969) indicated that insecticides taken up via physical contact spread over the cuticle and use the tracheae as portals of entry into the insect body. Sugiura et al. (2008) also suggested that the knockdown effect of directly applied oil-based aerosol was caused by the flow of pyrethroids into the mesothoracic spiracles, and their subsequent penetration through the inner wall of the mesothoracic tracheae.

In this study, our results suggested that spiracles are the most effective entry point for vaporized knockdown agents such as pyrethroids. The modes of insecticide entry into flying dipteran insects such as mosquitoes, tabanid flies, biting midges, blackflies, etc. are thought to be almost the same as those observed in houseflies. Our results might aid the optimization of formulations used for the control of insect pests of public health concern, as well as agricultural pests. For example, the enlargement of the integuments of bed bugs, *Cimex lectularius* L., has been reported to be one of the adaptations conferring insecticide resistance (Lehnert et al. 2011). The development of new formulations that assist in the entry of insecticides into the insect tracheal system might ultimately lead to the development of effective measures for the control of such resistant pests.
